# Exploring Syrian Refugees’ Access to Medical and Social Support Services Using a Trauma-Informed Analytic Framework

**DOI:** 10.3390/ijerph20032031

**Published:** 2023-01-22

**Authors:** Neda Moayerian, Max Stephenson, Muddather Abu Karaki, Renad Abbadi

**Affiliations:** 1 School of Urban Planning, University of Tehran, Tehran 14155-6619, Iran; 2 Institute for Policy and Governance, School of Public and International Affairs Virginia Tech, Blacksburg, VA 24061, USA; 3 Department of Media and Strategic Studies, Al-Hussein Bin Talal University, Ma’an 71111, Jordan; 4 Department of English Language and Literature, Al-Hussein Bin Talal University, Ma’an 71111, Jordan

**Keywords:** refugees, Syria, Jordan, trauma-informed framework, intersectionality, refugee camps

## Abstract

Even after arrival in new countries, refugees may be exposed to traumatic events. This state is exacerbated by contextual stressors, including the resettlement process, asylum proceedings and threats of deportation. This paper is rooted in a trauma-informed framework. We interviewed 16 male Syrian refugee migrant workers employed on a Jordanian farm during crop harvesting season to explore the quality and level of medical care and mental health services they received in light of the framework’s principal dimensions (e.g., safety, trust, intersectionality). We found that this vulnerable group of individuals is living a marginal and marginalized existence and depends on the goodwill of the growers for whom they work to treat them with a modicum of dignity and respect. Second, their itinerancy makes it difficult for this population to take advantage of available medical and mental health services at the nation’s major refugee camps. Finally, our interlocutors preferred their current lives, as isolating and limiting as they are, as superior to full-time residence in the camps, because they perceive their present way of life as according a measure of dignity, self-direction and autonomy they could not enjoy in the camps.

## 1. Introduction

The literature on refugees has burgeoned in recent years as the number of individuals meeting that definition has grown as social turmoil and civil or international conflict has engulfed several nations, including, among others, Syria, Yemen, Myanmar and the countries of the so-called Northern Triangle, Guatemala, Honduras and El Salvador, in Central America. This brutal reality has caused mass exodus from these countries and placed enormous stress on state and United Nations institutions to respond. A backlash to this tide of humanity has arisen in many affected nations, a share of whose leaders have sought to stigmatize and discriminate against these groups and to blame them for a range of issues occurring in those countries. Jordan had seen little of this sort of sort response until recently when attitudes among key government officials have publicly begun to harden.

Armed conflict in Syria began in 2011, resulting in massive, forced displacement of the Syrian population. As of 30 September 2022, there were 676,606 registered Syrian refugees residing in Jordan, 133,751 of whom live in camps [1]. Jordan has accepted approximately 1.5 million Syrian refugees (both registered and unregistered with the UNHCR) in recent years, a number that has stretched the country’s capacity to respond quite thin. In this context, it seems timely to explore how an especially vulnerable subset of an already susceptible population is now imagining its lived circumstances and prospects for moving forward within them. We recognize that doing so unfolds within a context in which, as Ostrander and colleagues have argued, “Compounding their initial trauma experiences, refugees often undergo further traumatic migration experiences and challenges after resettlement that can have long-lasting effects on their health and mental health” (2017, [2] p. 66).

Several authors have taken up this concern in refugee studies by focusing on the application of trauma-informed practice as a clinical intervention to decrease negative impacts of displacement among refugee youth [3,4,5] and adults [6] and as a tool to inform social work by documenting refugees’ traumatic experiences of [2,7]. However, to the best of our knowledge, there is no analysis that has adopted a trauma-informed framework to understand the relationship between sense of safety and peer support (determinants in the trauma-informed approach) and perceived individual capacity among displaced populations. We interviewed a group of quite vulnerable individuals during a time in which their rights and agency are very much under threat to determine how they view their level of safety, current support and collaboration as well as their own agency and efficacy. Such perceptions have proven to be critical to one’s capacity to pursue and/or to cooperate with efforts to secure change in the status quo. This article aims to deepen our understanding of that critical valence in refugee studies and policy and politics in a scenario that is severely testing the current international refugee regime.

### 1.1. The Trauma-Informed Perspective and the Study of Displaced Populations

Refugees and asylum seekers commonly experience displacement, persecution and substantial cultural adjustments, making them deeply vulnerable populations that deserve attention from helping professionals. This group often suffers multiple forms of trauma, “including lack of food or clean water, being displaced, lack of shelter, ill health without access to medical care, murder of a family member or someone known, being detained, and beatings” from their governments as well as from other contending forces [7]. In her research on Tamil refugees now residing in the United States or Canada, Weaver found that “most respondents reported dwelling on their traumatic experiences, feeling as though they were happening again, feeling hopeless, experiencing recurrent bad dreams, and having less interest in daily activities” (2016, [7] p. 120).

Even after arrival in new countries, refugees may continue to be exposed to traumatic events. This state is typically exacerbated by contextual stressors, including the resettlement process, asylum proceedings and threats of deportation for those without secure legal status [8]. In their study of a group of Syrians displaced by that nation’s civil war, Peconga and Høgh Thøgersen found that “[such] refugees could be over 10 times more likely to develop post-traumatic stress and other disorders than the general population” [9]. In their study of Syrian Refugees’ access to noncommunicable disease services in Jordan, McNatt and colleagues found that “Emotional distress is a central concern and is frequently highlighted as the trigger for a noncommunicable disease or its exacerbation” [10]. More broadly, Beiser and colleagues have contended that post-traumatic stress disorder (PTSD) “… militated against refugee economic integration” [11].

Although no population is immune to experiencing trauma, certain groups disproportionately experience certain forms of distress because of deeply entrenched structural inequalities [12]. George has built on refugee, post-colonial, trauma and feminist theories, and highlighted refugee suffering as “a consequence of multiple historical, social and political constraints which are embedded in the personal experiences of [those individuals]” [13]. Farmer and others have employed the concept of structural violence to link social inequalities with trauma and related anguish, arguing that “such suffering is structured by historically given (and often economically driven) processes and forces that conspire whether through routine, ritual, or, as is more commonly the case, these hard surfaces—to constrain agency” [14].

Sigvardsdotter and colleagues (2016) have reviewed existing studies addressing trauma among adult refugees in non-clinical settings and contended that the majority of those analyses have adopted checklists, “as a tool to control for background variables when studying refugee health, mental health in particular, [… this focus] has resulted in trauma checklists gaining less attention in their own right” [15]. To address this gap, we have adopted a trauma-informed care framework that “recognizes the intersection of trauma with many health and social problems for which people seek services and treatment, aiming to sensitively address trauma along with an individual’s issues” (2016, [12] p. 223).

We consider this focus timely since previous analyses have shown that despite calls for specialized mental health services, Syrian refugees in Jordan still suffer from trauma at different levels, with women more likely to develop PTSD symptoms and depression than men [16]. Moreover, access to mental healthcare is not equal across the Syrian refugee population:

[R]efugees in urban settings described more barriers to, and lack of information about services, than those in camps […] Women in particular, reported an increased need for privacy when accessing mental health services, for fear of experiencing stigma within the community.

[17]

In response to these needs, we explored the quality and level of medical care and mental health services our interviewees received in light of the trauma-informed framework’s principal dimensions (e.g., safety, trust, intersectionality).

### 1.2. Trauma-Informed Care Framework

United States government agencies (e.g., the U.S. Substance Abuse and Mental Health Services Administration) and researchers have outlined a set of core principles of trauma-informed care. While there is some variability in the terminology these have employed, there appears to be consensus in the literature that these tenets should include safety, trustworthiness and transparency, collaboration and peer support, empowerment, choice and the intersectionality of identity characteristics. We briefly describe each principle next.

#### 1.2.1. Safety

In the trauma-informed care framework, safety refers to the organization/program service users’ physical and emotional safety, meaning reasonable freedom from harm or danger and to prevent further shocks from occurring. UNHCR, as the major international refugee protection organization, faces difficult challenges in integrating these differing security interests and strategies in the contexts it routinely confronts.

The problems arising from operating in war zones and continuing protection concerns related to refugees in protracted situations are partly responsible for challenges in providing protection to individuals and defusing potential interstate tension. So too is the rise of xenophobia and fear of asylum seekers in many countries, which has led to a tendency to see refugees not as victims but as perpetrators of insecurity. That kind of thinking has inspired more aggressive interception measures, higher barriers to entry and indiscriminate detention, all of which pose new security risks to refugees [18].

**Figure 1:**
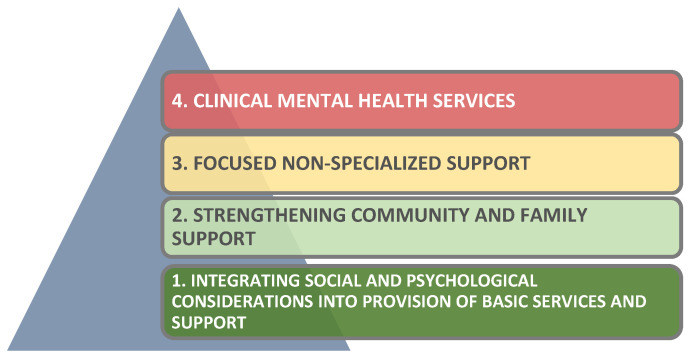
Multi-layered MHPSS services and supports (2021, [19] p. 3).

Aside from physical security, emotional/psychological safety plays a vital role in refugees’ well-being following displacement and one or more related traumatic experiences. UNHCR [19] personnel view refugee mental health and psychosocial support (MHPSS) as occurring on four levels (Figure 1):

Layer 1 (dark green in Figure 1) initiatives aim to provide basic services and security. Layer 2 (or light green in Figure 1) efforts seek to strengthen community and family support through promotion of activities that foster social cohesion and self-help. Layer 3 (yellow in Figure 1) assistance focuses on providing refugees emotional and practical aid through individual, family or group interventions. Trained non-specialized workers in health, education, community-based protection, child protection or sexual and gender-based violence (SGBV) usually deliver such support, with ongoing supervision. Layer 4 (red in Figure 1) offers clinical mental health and psychosocial services for those with severe symptoms or whose intolerable suffering renders them unable to carry out basic daily functions. Such interventions are usually led by mental health professionals, but also can also be offered by social workers (2021, [19] p. 4).

Examining the psychological functions of nostalgia among Syrian refugees residing in Saudi Arabia, Wildschut and others have contended that “Most established benefits of nostalgia [i.e., fostering self-continuity, a sense of meaning and self-esteem] also accrued to Syrian refugees. However, contrary to previous findings, [these authors found that] nostalgia decreased optimism, highlighting the limits of its palliative capacity among displaced individuals” [20]. Wildschut et al. found that individual dispositional resilience, “the capacity to withstand and recover from adversity”, acted as a catalyst of the nostalgic emotion’s benefits and, likewise, as an inhibitor of its costs [20]. Kalmanowitz and Ho have suggested that a combination of art therapy and meditation may “help individuals build strategies for safety, support resilience, and work with multiple levels of loss, after extreme and traumatic experiences” [21]. Afsharian and colleagues have contended that “fostering pathways to successful employment and creating safe work based on a high PSC [psychologically safe climate] and less harassment are strongly recommended to improve refugees’ mental health and adaptation” [22].

#### 1.2.2. Trust and Transparency

Trustworthiness and transparency refer to an organization’s openness in its policies and procedures, with the objective of building trust among constituent groups including staff, clients and community members. In their systematic review of articles reporting on trust amongst refugees in resettlement settings, Essex and colleagues [23] found that trust is relational, temporal and contextual. Since trust is relational, scholars have investigated various forms of it, including inter-or- intra-group [24], social, political, restorative and institutional [25], interpersonal trust between General Practitioners (GPs) and refugees [26] or between refugees and their sponsors in a host country [27]). Essex and his collaborators have contended this:

A major theme throughout the above articles was one of restoration, that is, amongst refugees and asylum seekers, trust had been lost or in some way damaged and that it was fundamental to re-establish this in resettlement. In addition to a range of past experiences, numerous factors were seen as important in the restoration of trust in resettlement. Generally, supportive individuals and institutions were seen as important foundations (2021, [23] p. 565).

In interviews with a group of asylum seekers in England, Hynes found that “Overall, the experience of being socially excluded and separated from mainstream services and, most importantly, the rights and entitlements of others left little space for the restoration of any institutional or political trust” [26]. Kyriakides et al. interviewed 204 refugees and developed the concept of “resettlement knowledge assets” and reported on “how these assets emerge through pre-arrival trust building, modify the resettlement expectations of both sponsors and sponsored, and reduce resettlement uncertainty” [27]. In their study of asylum seekers’ expectations of, and trust in, physicians and medical care providers assisting refugees in the UK, O’Donnell and colleagues argued that “[a]lthough asylum seekers were generally pleased with the care they received from the [National Health Service] NHS, […] GPs (general practitioners) and other healthcare professionals need to be aware that experience of different systems of care can have an impact on individuals’ expectations in a GP-led system” [26].

The UNHCR has developed a framework, “Accountability to Affected People (AAP),” to increase accountability in refugees’ protection, assistance and solutions interventions and programs. The four building blocks of the AAP framework include: participation and inclusion, communication and transparency, feedback and response and organizational learning and adaptation. The agency has defined transparency as “Women, men, girls, and boys of diverse backgrounds in all operations have access to timely, accurate, and relevant information on (i) their rights and entitlements, and (ii) UNHCR and its partners’ programs” [28]. However, past analyses have found a lack of transparency and external accountability among international aid organizations. For example, in a recent case, a UN special rapporteur contacted dozens of international organizations for details concerning their information transparency policies. Only a handful of entities—primarily financial institutions—responded [29]. For its part, the UN Secretariat, which lacks transparency standards across agencies and employs ad-hoc standards for access-to-information requests, did not respond (Kaye 2017, p. 4). Moreover, many other international organizations lack external accountability mechanisms, because news media do not subject them to the same “journalistic microscope” as national governments [30]. Crisis situations make such institutions’ efforts at transparency uniquely pertinent, yet their leaders often assert a false trade-off between operating transparency and easing human suffering as quickly as possible [31]. One study found, for example, that from the refugees’ point of view:

For many Congolese refugees, the most frustrating part of the resettlement process is the lack of transparency in how UNHCR representatives make decisions regarding their cases. Even UNHCR representatives have confided in me that they are frustrated by the lack of transparency within their own bureaucracy. In practice, resettlement procedures, like much humanitarian work, constitute a form of ‘adhocracy,’ the transformation of rational bureaucratic planning into guesswork and improvisation.

[32]

Or as Burkhardt and colleagues learned from their interviewees, female Syrian refugees in the Netherlands:

Many of the IND [Immigration and Naturalisation Service] regulations and criteria are not transparent and accessible to the refugees, who are at a disadvantage in understanding the implications of their choices or consenting to the various steps in the procedure. Confusion about technicalities, coding, acronyms used go together with the hazards of what gets lost in translation and the sensitive nature of intercultural communication that often leads to these women feeling intimidated, subalternized and agreeing to choices and ticking boxes that they barely understand.

[33]

#### 1.2.3. Collaboration, Empowerment and Choice

Collaboration in trauma-informed care demands that agency staff view service users as active partners and experts in their own lives, an approach often operationalized through the formal and informal use of peer support, including peer mentoring [34,35].

Empowerment efforts aim to share power with service users, encouraging their voice in decision making at individual and collective levels. “Empowerment” means different things to dissimilar groups and organizations. In in-depth interviews with refugee clients and organizational staff of two American refugee resettlement organizations, Steimel found that, “while organizational staff professed empowerment focused on self-sufficiency as self-determination, in practice their communication to clients defined self-sufficiency a priori in economic terms. Refugee-clients instead constructed empowerment(s) in economic, educational, personal, and family terms” [36].

In her 18-month assignment as Project Director for CARE International in Ngara, Tanzania, Benjamin documented major challenges regarding initiatives aimed at empowering Rwandan refugee women:

Women without partners were the least protected and took the greatest risks in their efforts to survive and feed their children. Their adaptive behavior increased their risks of rape, sexual abuse, and exposure to HIV and other sexually transmitted diseases. These serious problems were overshadowed by the chaotic business of running a refugee camp. In the rush to accommodate the influx of hundreds of thousands of refugees, the non-governmental organizations and UN agencies established a relief infrastructure that-perversely-gave the perpetrators of crimes positions of power within the camp, which enabled the gender violations to persist.

[37]

In a study of NGOs and refugee community organizations (RCOs) in Hong Kong, Lau found that the major challenges to empowerment of the displaced included principally “the financial dependence of mainstream NGOs on the government, the Hong Kong government’s perceptions of welfare policy and civil society, the existence of the international refugee regime and disunity among asylum seekers” [38]. Other research has suggested that having meaningful choice and options gives service users a level of control and is associated with better treatment outcomes in a variety of settings [39,40].

Overall, from a critical point of view, Pasha has argued:

A refugee camp is an ‘exceptional’ space existing outside the formal political order and the practical remit of rights; in them, refugees depend on the compassionate charity of strangers and lack avenues to enforce and realise their rights claims Seen from this perspective, expecting refugees to patiently endure a long-term liminal existence in camps, thus prolonging their dislocation from effective citizenship, only produces depoliticised and disempowered refugee subjects.

[41]

#### 1.2.4. Intersectionality

Finally, intersectionality implies awareness of identity characteristics, such as race, gender and sexual orientation, and the privileges or oppression these characteristics can represent [42]. In her Ph.D. thesis, Linn explored the lived experience of Syrian refugee women in Lebanon and Jordan. She found:

Whilst policies pertaining to Syrian refugees in Jordan and Lebanon are not gendered at the state level, in the micro day-to-day, the law shapes refugees’ experience in differentiated and gendered ways and has gendered consequences. This results in refugee women occupying a landscape of permitted or prohibited spaces based on policies and legal documentation, which intersect with structural issues of gender and patriarchy.

(2020, [43] p. 1)

The following section describes our methodology and how we employed a trauma- informed care framework in our data collection and analysis.

## 2. Materials and Methods

### 2.1. Case Study Research

We conducted 16 semi-structured interviews with members of a targeted group of Syrian refugees, now working as migrant workers and residing part of each year in Jordan near Ma’an and a portion in the Jordan Valley. These individuals receive a variety of support services from the nongovernmental organization Ma’an Orphanage Charitable Society (MOCS). In recent years, MOCS has sought to provide support and succor to the various populations of refugees residing in its service area. For the past decade or so, that group has included migrant agricultural workers (farmers) who fled Syria to avoid persecution or death as that nation’s civil war unfolded. We had hoped to interview the 16 male and female workers whom we had the opportunity to interview in September 2021 for another research project for this effort. However, as is the case with many work permit holders, they were not recruited to work at the same farm this year. In consequence, we wound up interviewing a fresh group of workers for this study.

### 2.2. Recruitment

One of the authors, Dr. Karaki, worked with the Director of the Ma’an Orphanage Charitable Society to contact possible interviewees for this inquiry and to inform those individuals of the study team’s interest in speaking with them to discuss their experiences as itinerant workers. We assured each of our study participants that we would work to protect the confidentiality of their responses by assigning each a pseudonym and we have done so here by identifying individuals only with a number.

Drs. Karaki and Abbadi of our study team conducted the interviews in Arabic at the farm near Ma’an at which our interlocutors were working. This occurred at the request of the landowner/grower who, while willing to allow each individual time to participate in an interview, was unwilling to have them take additional time away from crop harvesting to travel to that city and return for the purpose of the interview.

### 2.3. Interview Process and Ethics

Our team’s Jordanian scholars served in this interviewing role, too, because of the fluency of each in the local language and the existence of travel difficulties from the United States and Iran linked to the COVID-19 pandemic for Drs. Stephenson and Moayerian. The team also believed that the interviews might go more smoothly, and our interviewees prove more willing to share their views and perceptions, in the absence of obvious foreigners (Stephenson and Moayerian). Based on the 6 criteria of the trauma-informed care framework (i.e., safety, trustworthiness and transparency, collaboration and peer support, empowerment, choice and the intersectionality of identity characteristics) we devised 8 semi-structured questions with which to approach our 16 interviewees (provided in Appendix A) and estimated that each conversation would take perhaps 45 min.

The team had hoped to record our interview sessions and transcribe them verbatim, but it became clear that our potential respondents were concerned not to say something that might redound to harm themselves or their families, despite our aim to protect their confidentiality. Most had good reason not to trust authority, given their experience under the autocratic and abusive Assad regime in their home nation. Accordingly, we shifted course and Dr. Karaki conducted each interview, with Dr. Abbadi taking as close to literal notes of each response as feasible. Dr. Abbadi is a professional linguist and she recorded contemporaneous notes for each response and interview in English so that all members of the team could consider and code individuals’ replies and review the interviews as a group as well. To ensure the reliability of data, after each question, Dr. Abbadi described the notes she wrote so the participants and the interviewers each had an opportunity to confirm their accuracy. In the consent form, we informed our interviewees that they could review the interview notes following their interview, if they wished. However, our interviewees found the simultaneous note-checking process satisfactory and did not request to do so.

Similar to most qualitative inquiries, we did not expect, or conclude, that the findings from this study would be generalizable in a statistical sense, but we are and were hopeful that our arguments, rooted in thoughtful theorization, might help illuminate the character of the continuing challenges confronting refugees in an international context organized on the principle of state sovereignty and animated by the relentless capacity for othering exhibited by human populations across the globe. In this sense, we hope our findings may be analytically generalizable.

### 2.4. Deductive thematic Analysis

We followed Braun and Clarke’s [44] method for our theoretical/deductive thematic analysis of the interviews. Team members independently coded, that is, conducted a content/thematic analysis of the interview transcripts as our principal source of data, and we thereafter discussed our conclusions amongst ourselves until gaining consensus. The themes the team identified included safety, trust and transparency; collaboration, empowerment and choice; and intersectionality with emergent subthemes, such as living conditions, legal status concerns, social relationships and employment and rights awareness.

**Figure 2:**
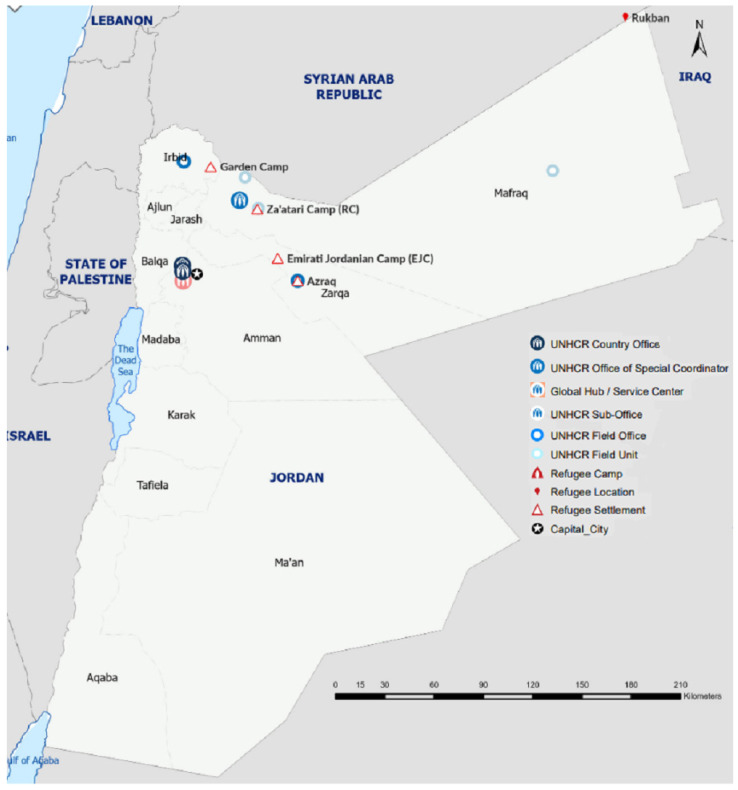
Location of the refugee camps and Ma’an in Jordan [46].

All the interviewees formally resided in a UNHCR camp located in northern Jordan and had been granted a monthly permit by the Jordanian government to leave the camp and work—harvest vegetables—on the farm where our team members spoke with them. (Refugees residing in camps can obtain a work permit free of charge to work across the country in occupations open to non-Jordanians. The permit serves as a one-month leave/license, facilitating movement in and out of a camp [45].) The refugees/workers must travel once per month during the harvest season to obtain a renewal of their work permits. During the winter, they either return to the camp or, more frequently, work on farms in the Jordan Valley nearby. Figure 2 shows the location of the UNHCR camps and Ma’an in Jordan [46].

Formal residence in the camps entitles refugees to medical services, monthly support provided by the UNHCR and/or any other international aid services afforded by the Jordanian government. The Al Ma’an charitable society provides access to medical and educational services by covering costs at the clinics and schools in Ma’an for those refugees with work permits or those who are residents. Additionally, the NGO distributes food supplies, clothing and other forms of in-kind support to the refugees it serves. 

Syrian refugee children who live in a UNHCR camp have access to school there. Outside the camps, refugee youths attend classes in the afternoon session in nearby government schools. The Jordanian government has developed a special program for refugee youngsters designed to support their education without overwhelming its educational system and resources. 

## 3. Results and Discussion

### 3.1. Study Population

Table 1 provides an overview of some of the most salient characteristics of our interviewees.

In brief, as a group, our interviewees were relatively young, averaging 41.5 years of age, and were supporting comparatively large families comprised of an average of 5.6 members.

### 3.2. Themes 

#### 3.2.1. Safety

Most of the interviewees (14 of 16) considered their current situation safe(er) in Jordan than it would be in Syria:

[Before the civil war] we lived in safety despite the economic situation, but because of the conflict, we decided to leave Syria out of fear for my family. It was an unsuccessful experience to move inside Syrian territory until we reached the Jordanian border.

(Interviewee, 2, 11 September 2022)

Many of the interviewees regarded travel within Syria as one of the most traumatic experiences they had in their journey: “Traveling in war zones inside Syria was the biggest shock, as we witnessed destruction and murderers everywhere. It was a journey full of fear and terror” (Interviewee, 3 September 2022).

On 10 August 2020, Jordanian authorities forcibly transferred at least 16 Syrian refugees, including eight children aged between 4 and 14, to an informal camp in a no man’s land located in the desert between Syria and Jordan [47]. Nine of the interviewees explained that psychologically they do not feel safe in their refugee status, living far from their families who reside in camps or in Syria and leaving their home/land behind; “Physically and emotionally I feel it [safety], but psychologically it is not possible to be outside your country, forced out because of the war. It is not possible to be psychologically comfortable” (Interviewee, 7, 17 September 2022). Or, as interviewee 8 shared with us,

I do not feel safe because of our situation as a refugee and being far from family and relatives, despite all the services and assistance, nothing can compensate for your homeland, but because of the conditions of war and the death of my wife, we decided to leave [Syria].

(Interviewee, 8, 17 September 2022)

Ten interviewees mentioned living in a camp as traumatic/problematic. In response to “what are some conditions that can lead to a more fulfilling life for you and your family whether you decide to return to Syria or to stay in Jordan or other countries?”, many mentioned the improvement of their living situation in camps and/or having an independent place to live.

Previous studies have also highlighted living in camps as one of the major stressors resulting in PTSD among refugees. Braun-Lewensohn and Al-Sayed [48] found that the amount of time spent in a refugee camp, gender and exposure to war situations contributed to the explained variance in psychological problems of camp residents. While many refugees hope to leave the camps one day,

With the bailout process suspended, there is presently no legal way for refugees residing in the camps to leave permanently in order to settle in host communities, save for limited cases approved by the government Humanitarian Committee in the camps, which may include family reunification, medical cases or other vulnerable profiles.

[49]

Even if the bailout process had not ended, it seems rather unlikely that our interviewees had the support to benefit from it:

The ‘bailout’ system [a process through which Syrians who had identified a Jordanian sponsor were able to leave the camps and settle in urban areas] has an important class element as it has often enabled those Syrians with sufficient access to capital and connections to leave refugee camps and move into Jordanian host communities, but has simultaneously effectively consigned to camps the poorest Syrians ….

[50]

Unlike the living conditions, all the interviewees considered their access to medical services at the camp as above satisfactory especially concerning addressing the medical needs of children and women; “We got medical services, and it happened to my mother, my wife, and the children. We had two children in the camp, and we got all their needs covered, thank God” (Interviewee, 11, 19 September 2022). Given the fact that the average age of our interviewees was 41.5 years old, their level of satisfaction with medical services nonetheless appeared to align with other studies’ findings involving different age groups, “Of the 500 participants, the satisfaction rate was 72.5%. Young participants and participants with a shorter stay in the camp showed higher overall satisfaction rates [with healthcare services]” [51]. While most of the interviewees mentioned regular/monthly physical examinations and some alluded to the comprehensive medical exam they received at their initial border crossing, none of our respondents discussed receiving mental health services except for interviewee 6 who argued, “regarding the mental services, we do not need them. Things are better from the inside” (Interviewee, 6, 14 September 2022).

Al-Soleiti and colleagues have investigated the barriers to Syrian refugees’ access to mental health services in Jordan and contended

In collaboration with the UNHCR and numerous NGOs, a wide spectrum of mental [health] services are offered and continue to grow. Significant barriers remain to improving accessibility of mental health services for Syrian refugees in Jordan, including financial limitations [both organizational budgetary limitations and the financial hardship of refugee patients], transportation difficulties, clinician shortages and burnout, inflexible organizational policies, treatment stigma, limited or absent screening protocols, and security restrictions in high-security settings.

[52]

The narratives of our interviewees, who have experienced living in camps and in communities for parts of each year, highlighted the fact that living in camps, the unpredictable situation of refugee status and concerns for left-behind family members are all post-migration stressors that limit adaptive coping strategies among refugees. Similar to Thorleifsson’s findings concerning Syrian refugees in Lebanon, our interviewees also sought to address these stressors in part, “by engaging in the unskilled labor market (agriculture and construction), which provides low and insufficient incomes […] and forming social relationships with other refugees” [53].

#### 3.2.2. Trust and Transparency

We asked the interviewees whether in their current situation they trust their colleagues, bosses and people around them. Most of the interviewees indicated that they trusted their colleagues: “There is no room for mistrust, as we are the same, my colleagues and I suffer from the same situation” (Interviewee, 1, 10 September 2022). However, a few interviewees noted some limitations: “Yes, I trust them, but within the limits of work and fellowship” (Interviewee, 13, 20 September 2022), or, as 7 put it, “There is trust in them, but not in everything, out of fear of where the refugees will be watching us. Therefore, I deal with colleagues with caution, and the mentors, my dealings with them are limited” (Interviewee, 17 September 2022).

As noted above, in most situations, refugees shared some level of mistrust of others and not being trusted. In our case, one of the reasons behind a comparatively high level of trust among the interviewees seems to have resulted from the similarity of language and culture between the host and guest nation residents. All the interviewees indicated that they had no problem understanding the language or other cultural factors: “The same language, customs, traditions and the Levant (Levant, (from the French lever, “to rise,” as in sunrise, meaning the east), historically, the region along the eastern Mediterranean shore, roughly corresponding to modern-day Israel, Jordan, Lebanon, Syria, and certain adjacent areas [54]) [culture] share these factors, so it was a better experience than moving to Turkey because of the language” (Interviewee, 1, 10 September 2022). Not needing a translator facilitates communication and reduces uncertainty between both groups.

A refugee may also feel more reluctant to participate in meaningful communication if the interpreter present is not trusted. Given ethnic or social class differences, the interpreter may have (or be perceived to have) biases that would influence their translation and decisions taken as a result [55].

Another reason for their expressed level of trust may have arisen from the fact that our interviewees were employed and had constant communication with their Jordanian employer. As Fajth and colleagues’ found concerning Congolese refugees in Rwanda, “greater (economic) interaction between the two populations helps increase trust between refugees and host communities over time” (2019, [56] p. 1). Similarly, Tobin and colleagues have found that, in Jordan, “The refugees’ relations with the host populations are amicable but distant; often, the relations grow stronger through trust-based economic ties and conflict resolution”(2021, [57] p. 23).

All of those we interviewed viewed the procedures governing their refugee status as clear and straightforward: “I am illiterate, but officials were welcoming at the camp and explained to us the rules of staying at camp. We had to sign forms, but everything was read out to us” (Interviewee, 1, 10 September 2022). From the unanimity of responses regarding officials’ welcoming behavior, we are persuaded that trauma-informed training workshops haven proven effective. For example, in 2017, the UN International Organization for Migration provided four psychosocial support training sessions (3 days each) to frontline officers to improve the capacity of frontline protection and health workers to incorporate mental health and psychosocial support in their work with Syrian refugees.

Once again, one can see the impacts of similar language between the host and guest countries in improving process transparency. Another potential factor arises from the fact that most of those who are registered with the UNHCR, i.e., formally reside under its auspices, hold Asylum Seeker Cards (ASC) and only a few Syrian refugees undergo the Refugee Status Determination (RSD) process, which has been described as “frustrating” due to “the lack of transparency in how UNHCR representatives make decisions regarding their cases” ( 2012, [32] p. 198).

#### 3.2.3. Collaboration, Empowerment and Choice

We previously interviewed 16 male and female Syrian refugees who worked on the same farm in September 2021. None of the 32 interviewees with whom we have spoken to date were conversant concerning the human rights agreements, such as the 1951 Geneva Convention, that govern refugee rights as a matter of international law, or of Jordan’s laws that affect refugees. Facilitating access to institutions and organizations, such as national human rights entities that can help raise awareness among this population, might constitute a useful first step in shifting their level of knowledge of their rights and responsibilities.

A few of our interviewees mentioned educational workshops for refugees to set up “small projects.” Even if these initiatives do not immediately translate into lucrative businesses securing those individuals’ economic self-reliance, as Pasha has argued,

refugees may gain ‘social capital’, including new skills and certification through their participation in specialised training courses [linked to such efforts]. These skills, qualifications and dispositions may eventually translate into real empowerment for some in the future beyond the confines of the camp.

(2020, [41] p. 256)

All our interviewees were farm workers and had gained some level of economic independence through their vocation. However, comparing their situation to that of the time when they worked on their own lands led several of them to indicate they now felt less in control of their lives: “the difference was that we worked on our lands and because we worked for wages” (Interviewee, 2, September 11, 2022). All the interviewees had lived in a camp for at least three years when we interviewed them. Ethnographic research across contexts has found that expressions of political agency in the refugee camps, including the formation of self-governance structures and protests for greater rights, are often not tolerated and can lead to deportation and other punitive measures [58]. Such could be the case for our interviewees, as well, since many of them mentioned their hope to settle outside of the camps as soon as they could do so.

When asked “what are some conditions that could lead to a more fulfilling life for you and your family whether you decide to return to Syria or to stay in Jordan or move to another country?”, most of the interviewees referred to two issues. First, longer work permit renewal times to avoid monthly trips to the camps and, second, the establishment of democratic governance structures in Syria. The first hope addresses a disempowering/limiting policy affecting refugees in their current situation, while the second aim envisions a freer society that enables its citizens to exercise their agency in pursuing their life goals.

#### 3.2.4. Intersectionality

The majority of the intersectionality and trauma literature regarding Syrian refugees has focused on issues that women and LGBTQ individuals have confronted in their host countries [43,59,60,61,62]. As we note below, we were not able to interview female farm workers and doing so might have shed light on certain cultural structures intersecting with gender roles among our interviewee families. To investigate the trauma-informed framework via the lens of intersectionality, we focused on the males with whom we had an opportunity to speak. We consider class, gender and legal status intersectionality briefly here. Hancock [63] has defined class in intersectionality as a “fuzzy” concept that not only includes economic income indicators, but also the social backgrounds of individuals including their educational achievements and the “cultural capital” they acquire through their families and living environments. She has contended that this broader-gauged socioeconomical profile better captures the “class” identity of individuals. Stave and Hillesund [64] have compared the Syrian refugees who fled to Lebanon to their counterparts in Jordan in such terms. On average, they found the latter group was less economically advantaged, more rural in origin and less well-educated than its host population. In our case, our interlocuters were all farmers prior in Syria and one of them mentioned he was illiterate. The intersection of class and legal status has forced our interviewees to live in camps despite their expressed desire not to do so. Wealthier refugees benefitted from the bailout system between 2014–2015 and could leave the camps and reside in Jordan’s cities, but none of our interviewees had the means to participate in that process.

Another vulnerability for those with whom we spoke arose at the intersection of class, legal status and gender. All our interviewees self-described as male. Nielsen found in research concerning Syrian refugees in Turkey that “the Syrians who worked in these jobs [i.e., construction, agriculture, and other low-skilled labor] were still grateful to be able to provide for their families” even in the face of labor vulnerabilities [65]. Scholars have attributed this behavior to one of the codes of masculinity, the “ideal man” as breadwinner, which is considered to be stress-provoking [66]. Moreover, past research has suggested that refugees with restricted access to economic opportunities, as a result of limited work rights and employment prospects, such as those our interviewees confront, who work seasonally and with temporary work permits, have worse mental health outcomes compared to those with greater access to economic opportunities [67]. In this regard, we noticed considerable anxiety among our interviewees to return to work (crop harvesting) as soon as possible, even though they had been granted permission to spend an hour with our team. Several of the interviewees also mentioned the anxiety provoked by their role as heads of households: “Yes, I feel safe, but the working conditions and the presence of my children in the camp and [the fact that] I am far from them [as I work], makes me feel afraid for them” (Interviewee, 9, 17 September 2022) or “I feel safe for myself, but because of the presence of my mother and sisters, being the only young man, I feel a kind of fear for them” (Interviewee, 10, 17 September 2022). The fact that the interviewees continue to work on the farm despite their low wages, the significant cost and inconvenient process of renewing their work permits suggests the vulnerability they experience created by the intersection of their gender, class and legal status.

### 3.3. Limitations

Although both male and female refugees were working on the farm, only male interviewees consented to participate in an interview. They asked us not to speak with their female partners to protect their families. We consider this one of the limitations of this study since, as previous research has shown:

Children and women refugees are considered to be more at risk than men in terms of psychological and sexual violence, first of all if they belong to female headed households. Child labour and early marriage are acknowledged as existing phenomena among Syrian refugees, although they are not fully perceived as something negative.

(2012, [68] p. 3)

Another possible limitation of our effort arose from the fact that our interviewers were both from Jordan, and since Syrian refugees, like other members of displaced populations, do not wish to seem ungrateful to their host nation or its residents, it is possible that our interviewees avoided sharing the severity of their challenges as a result.

## 4. Conclusions

The Syrian refugees we interviewed, having been displaced from their homes by war, have now lived from 4 to 6 years in situations of insecure legal status, low wages, ongoing concerns for their loved ones and little hope of returning to their past lives. We have explored whether and in what ways the vulnerable migrant refugees we interviewed were receiving comprehensive trauma-informed care and support to help to address this ongoing scenario. While our interviewees unanimously suggested they were satisfied with the content, quality and frequency of the medical services they have received, we did not find evidence that those we interviewed were receiving mental health services or specific support to address any continuing trauma. Bawadi and colleagues’ [69] recommendation to “introduce mental health services to the primary healthcare service” for refugee populations proved relevant to the population with whom we spoke.

In terms of collaboration, empowerment and choice, which we consider to be interconnected, the basic needs approach of the UNHCR and other primary service providers has only partially supported the economic independence of our interlocuters. While it seems clear that the provision of such services can encourage refugees to exercise and realize some level of individual agency, it is only through deliberate steps, including raising awareness among the refugees themselves of their rights and providing opportunities aimed at enhancing their capacities for their meaningful participation in decisions concerning the factors that most influence their lives, that empowerment at the individual and collective level for this vulnerable population may be achieved. Meanwhile, they will continue to live within a context of continuing trauma, displacement and economic and social vulnerability.

We can offer perhaps three additional final observations. First, this vulnerable group of individuals is clearly living a marginal and marginalized existence. It takes nothing away from the charity and goodwill of Jordanians and the Kingdom to observe that these farmworkers depend overwhelmingly on the good will of the growers for whom they work to treat them with a modicum of dignity and respect. They toil in positions that the government has declared Jordanians do not otherwise wish to pursue and they do so while living in/near the fields they are tending. They possess little and labor arduously for the wages they are provided. One must hope those are fair and justly provided. It was clear our interviewees understood their essential precarity as they sought to minimize the time away from their task, even though the owner of the property at which they were working had granted them time to speak with us. Second, their itinerancy makes it especially difficult for this population to take advantage of such mental health services as are available via the camps, since they can only be away for limited periods and must travel to those locations principally to renew their work permits and receive “essential” medical support. Finally, our interlocutors preferred their current lives, as isolating and limiting as they clearly are, being superior to continued full-time residence in the camps, which they saw as soulless and demeaning. That is, while plainly poor and plainly psychologically hobbled by possessing no real sense of when or whether they might be able to return home or to gain an alternate position in the society now hosting them, they nonetheless preferred their current circumstances precisely because those accord them a measure of dignity, self-direction and autonomy, however bounded, they perceive they could not enjoy in the camps.

## Figures and Tables

**Table 1:** Interviewees’ Demographics.

Interviewee	Age	Family Size	Al Ma’an Membership	Interview Date
1	25	3	5 years	10 September 2022
2	47	6	6 years	11 September 2022
3	57	8	Not reported	11 September 2022
4	62	9	6 years	13 September 2022
5	64	5	5 years	14 September 2022
6	39	5	4 years	14 September 2022
7	42	5	6 years	17 September 2022
8	57	6	6 years	17 September 2022
9	43	5	6 years	17 September 2022
10	36	5	6 years	17 September 2022
11	32	6	5 years	19 September 2022
12	26	4	3 years	19 September 2022
13	31	6	4 years	20 September 2022
14	36	5	5 years	20 September 2022
15	42	7	6 years	20 September 2022
16	25	5	4 years	20 September 2022

## Data Availability

Not applicable.

## Appendix A

### Interview Protocol & Questions

Date

Pseudonym

**Introduction by Interviewer(s)**

- Introduce self or selves
- Discuss the purpose(s) of the study
- Ask if they have any questions regarding the consent form or the research
- Present the structure of the interview

**Introductory Questions**

- How old are you?
- How long is it you are a member of Ma’an Orphans Charitable Society?
- How did you hear about this organization?

**Substantive Questions**

Intro: I am going to ask you some questions about your experience as a refugee since the time you left Syria, because I am interested in learning more about the level of support and security available for those who have to flee their countries. I hope to understand, in your view, if and how strong support systems for refugees and refugee’s capacity to continue a fulfilling life (either in their home or a host country) are connected.

1. Are you currently feel safe and secured physically, psychologically and emotionally?
  If not, can you share with us what hinders your feeling of safety/security?
2. Since you left Syria, have you experienced re-traumatization due to certain questions/processes? Can you share what can be done to avoid such incidents?
3. In your current situation, do you trust your colleagues, bosses and people around you?
4. Do you feel processes related to your refugee status and work are well explained and transparent?
  Can you share with us if there are points that can increase transparency of procedures for you?
5. Have you been in touch with any medical professionals to assess your potential physical and/or mental needs since your arrival in Jordan?
  If yes, how did you find the experience?
  Was the medical professional also a (Syrian) refugee?
6. Can you share with us, since your departure which organizations/individuals have tried to assist you in your resettlement journey and in which ways?
7. Thinking about the care you (should) have received thus far, how culturally (e.g., language/customs/food and etc.) relevant they have been? What steps can be done to make such efforts more effective and relatable to individuals receiving them?
8. In your view, what are some conditions that can lead to a more fulfilling life for you and your family whether you decide to return to Syria or to stay in Jordan or other countries?

**Concluding Questions**

- Is there anything else you would like to share concerning your experience with refugee care systems that you believe is important for me (us) to know?

**Wrapping Up Interview**

- Thank them for their time and participation
- Offer to send them interview notes to make sure we captured their ideas and comments accurately
- Inform them that their interview will be translated to English, and that their identity will remain confidential–we will use pseudonyms
- Record observations, thoughts, and/or reactions concerning the interview in field notes.
